# A Currency for Offsetting Energy Development Impacts: Horse-Trading Sage-Grouse on the Open Market

**DOI:** 10.1371/journal.pone.0010339

**Published:** 2010-04-28

**Authors:** Kevin E. Doherty, David E. Naugle, Jeffrey S. Evans

**Affiliations:** 1 National Audubon Society Science, Wyoming Audubon, Laramie, Wyoming, United States of America; 2 Wildlife Biology Program, University of Montana, Missoula, Montana, United States of America; 3 North America Science, The Nature Conservancy, Fort Collins, Colorado, United States of America; University of California, Berkeley, United States of America

## Abstract

**Background:**

Biodiversity offsets provide a mechanism to compensate for unavoidable damages from new energy development as the U.S. increases its domestic production. Proponents argue that offsets provide a partial solution for funding conservation while opponents contend the practice is flawed because offsets are negotiated without the science necessary to backup resulting decisions. Missing in negotiations is a biologically-based currency for estimating sufficiency of offsets and a framework for applying proceeds to maximize conservation benefits.

**Methodology/Principal Findings:**

Here we quantify a common currency for offsets for greater sage-grouse (*Centrocercus urophasianus*) by estimating number of impacted birds at 4 levels of development commonly permitted. Impacts were indiscernible at 1–12 wells per 32.2 km^2^. Above this threshold lek losses were 2–5 times greater inside than outside of development and bird abundance at remaining leks declined by −32 to −77%. Findings reiterated the importance of time-lags as evidenced by greater impacts 4 years after initial development. Clustering well locations enabled a few small leks to remain active inside of developments.

**Conclusions/Significance:**

Documented impacts relative to development intensity can be used to forecast biological trade-offs of newly proposed or ongoing developments, and when drilling is approved, anticipated bird declines form the biological currency for negotiating offsets.

Monetary costs for offsets will be determined by true conservation cost to mitigate risks such as sagebrush tillage to other populations of equal or greater number. If this information is blended with landscape level conservation planning, the mitigation hierarchy can be improved by steering planned developments away from conservation priorities, ensuring compensatory mitigation projects deliver a higher return for conservation that equate to an equal number of birds in the highest priority areas, provide on-site mitigation recommendations, and provide a biologically based cost for mitigating unavoidable impacts.

## Introduction

Species are disappearing and ecosystems are being degraded at alarming rates around the world [1]. Extinctions are largely the result of human activity [2], and investment in developments to support continued growth and prosperity makes evident the conservation challenges ahead. Biodiversity offsetting, also known as conservation banking, off-site mitigation, and habitat set-asides, are designed to compensate for unavoidable damages to wildlife populations from development [3]. The concept of offsets, widely used to mitigate wetland losses since the 1970s [4], has gained popularity around the world as a solution to other natural resource issues [5]. Offsets by definition are additional to other measures within the mitigation hierarchy put in place to avoid, or minimize environmental damage, and were never intended to replace responsible land stewardship [6]. A ‘hopeful but cautious optimism’ might best characterize enthusiasm for the concept of biodiversity offsets as a means of raising awareness of biodiversity costs and as a mechanism for funding large-scale conservation [6], [3], [7]. Evaluations of offset programs in other systems are mixed because success was judged by protecting, enhancing or restoring elsewhere a ‘like’ amount of habitat; but sufficiency of offsets requires a more reliable currency than habitat area [3].

Negotiating offsets may be akin to horse-trading on the open market; but science can help inform the biological basis for negotiations to ensure that the true benefits to conservation are realized. Offset proponents contend that industry is afforded the opportunity to add biodiversity costs into their balance sheets, but opponents argue that the practice is flawed because offsets are negotiated without the science necessary to backup resulting decisions [7]. Missing in negotiations is a biologically-based and common currency for estimating sufficiency of offsets.

Global energy demand increased by 50% in the last half-century and a similar increase is projected by 2030 [8]. The idea of offsets has surfaced as one tool to mitigate biodiversity impacts resulting from energy development [9]. Demand for domestic oil and gas resources portends the severity of future losses because extraction impacts wildlife directly by altering habitat use [10] and population dynamics [11], and indirectly by facilitating spread of invasive plants [12] and exotic diseases [13]. Creative solutions will still be needed even if renewable energy meets 20% of U.S. demand [14] because negative impacts of wind developments on wildlife are already evident [15].

Recent work has blended landscape level conservation planning with the mitigation hierarchy as a way to balance energy development with conservation values [16]. The strategy under Energy by Design (EbyD; [16]) is to improve approaches to the mitigation hierarchy (Avoid, Minimize, Restore, Offset,), steer planned developments away from conservation priorities, ensure compensatory mitigation projects deliver a higher return for conservation, and mobilize funding for conservation. Greater Sage-grouse (*Centrocercus urophasianus;* hereafter sage-grouse) are a focal species of high conservation concern which have been petitioned for the Endangered Species Act 9 times and are under a current listing petition decision. Sage-grouse also serve as a prime example of the landscape level analyses phases of EbyD. Human impacts within the last century have resulted in loss and degradation of sagebrush ecosystems in western North America [17]. Expanding oil and gas developments represent the newest stressor that exacerbates ongoing conservation challenges in this system [11], [18], [19], [20], [21]. A framework for conservation planning has been developed to evaluate options for reducing development impacts on sage-grouse in Wyoming, Montana, Colorado, Utah, and North and South Dakota [22]. Analyses showed that by selecting highest density areas first, managers could define core regions that contained 25, 50, 75, and 100% of the breeding population within 5, 12, 30, and 60% of the eastern sage-grouse range, respectively.

Identification and mapping of core-regions provided the mechanism for assessing trade-offs between biological value and anthropogenic risk to deliver the greatest conservation benefit to populations [23], [24], [25], [22]. Examination of conflicts between development potential and biological value gave insight into where specific landscapes fell within the mitigation hierarchy [22]. Ecological zoning of this nature is an admission that threats are large, resources are limited, and conservation action targeting every remaining population is unfeasible. Core regions represent a proactive attempt to identify and maintain a viable set of populations before the opportunity to do so is lost and can direct conservation to where actions will have the largest benefit to populations.

Still, missing in the core region strategy and in project level mitigation planning within EbyD is a rigorous, biologically based common currency for estimating residual impacts of energy development. The goal of this study was to create a currency for establishing offsets. We hypothesized that lek abundances and rates of inactivity are a process of well density when compared to a control populations outside of areas being developed for energy production. We then propose the resulting matrix of sage-grouse responses can be used as a currency for evaluating offsets rather than habitat area. This common currency is essential to mitigate residual impacts that cannot be avoided to ensure offsets alleviate threats elsewhere that would otherwise impact an equal or greater number of sage-grouse. We put our findings into context by discussing how offsets should reflect costs required to reduce future anticipated impacts. Lastly, we recommend an approach for integrating offsets into landscape planning by guiding offsets to priority landscapes where the greatest conservation benefits can be realized.

## Methods

### Study area

We conducted this study in Wyoming, a state central to sage-grouse conservation, representing >25% of the range-wide population [26] and 64% of the known population in their eastern range [22]. Extensive energy developments in Wyoming also provided the full range of impacts necessary to create a reliable currency for establishing offsets.

### Biological currency

We used lek count data to test for differences in rates of lek activity and bird abundance at five levels of energy development. These estimates form the basis of our currency for establishing offsets. Estimates are valuable in negotiating offsets because average rates of lek loss and declines in birds at remaining leks can be applied to any ongoing or newly proposed development to predict anticipated impacts. Lek count data is a reliable index to relative abundance that is used by agencies to monitor trends in sage-grouse numbers [27]. State, federal and contract employees count the number of displaying males at each known lek throughout Wyoming. Leks are typically counted in early morning ≥3 times in spring. Lek count protocols are available in the Wyoming Greater Sage-Grouse Conservation Plan [28]. We obtained lek count data from Wyoming Game and Fish, the state agency responsible for maintaining this public database.

We analyzed all leks in Wyoming so that findings apply to the types and levels of energy development common to sagebrush ecosystems in the West. We quantified impact of energy development on sage-grouse to form a biological basis for mitigation planning by testing four predictions 1) risk of lek loss was higher inside than outside of areas being developed for energy production, 2) bird abundance was lower at leks that remained active inside than outside of areas being developed for energy production, 3) rates of lek loss and bird abundance were related to levels of development commonly permitted in the landscape, and 4) time-lags influenced lek inactivity or bird abundance inside versus outside of areas being developed for energy production.

We used maximum counts of males from 1997 to 2007 at active (*n* = 1,190) and inactive (*n* = 154) leks. We classified a lek as active when each of three criteria were met: 1) ≥5 males counted at least once in 11 years, 2) ≥2 males counted in 2 different years, and 3) ≥2 males counted in one of the last three years [29]. The third criteria helped maintain sample sizes because each lek is not counted every year but most are counted at least once every three years. If a lek was active in 2005 but was not surveyed again in 2006 or 2007 we presumed it remained active. We classified a lek as inactive if it met the first two criteria but had zero males counted in the last year surveyed and was located >2.5-km from an active lek. The last criterion reduced bias in rate of lek loss by excluding from analyses the status of satellite leks whose formation and fate is typically tied to that of a larger nearby lek [26]. We used maximum number of males counted in 2007 at active leks (*n* = 1,035) to test if bird abundance was lower inside than outside of development. Number of active leks is reduced in this analysis because all known leks were not counted in 2007.

### Development mitigation categories

We made our analyses relevant to natural resource managers by defining non-arbitrary oil and gas development density categories that correspond to how development fields are permitted. The maximum number of wells in each category was used to define levels of development (oil and gas well spacing) that are commonly permitted on public lands. We used number of wells within 32.2 km^2^ (3.2-km radius) of a lek to classify each lek into one of five categories of energy development [30], [19]. Category 1 included control leks with no wells within 32.2 km^2^. Categories 2–5 represented increasing levels of development. Category 2 tested for impacts at 1–12 wells within 32.2 km^2^ (∼1 well per section [259 ha or 640 ac]) a level of development that is recommended by agencies to avoid impacts to sage-grouse. Category 3 tested for impacts at 13–39 wells (65 ha or 160 ac spacing), a level of development below what is permitted on public lands. Category 4 tested for impacts at 40–100 wells (32 ha or 80 ac spacing), a level that is commonly permitted on state and federal lands. Category 5 tested for impacts at 101–199 wells (16 ha or 40 ac spacing), a level of development that is common on private lands and is allowed by special permit on some federal lands. We excluded from analyses 1 outlier with >199 wells within 32.2 km^2^ that was still active.

We obtained well locations (*n* = 54,369) from the Wyoming Oil and Gas Conservation Commission 15 February 2008 and selected well that were in the ground by 1 March 2007. We excluded from analyses approved permits for wells that had not yet been drilled, plugged and abandoned wells, and 121 well locations that lacked a status code. We included in analyses wells (*n* = 33,275) that were in the ground by 1 March 2003 to test for time lags. We included wells that had not been plugged and abandoned by 1 March 2003.

**Figure 1:**
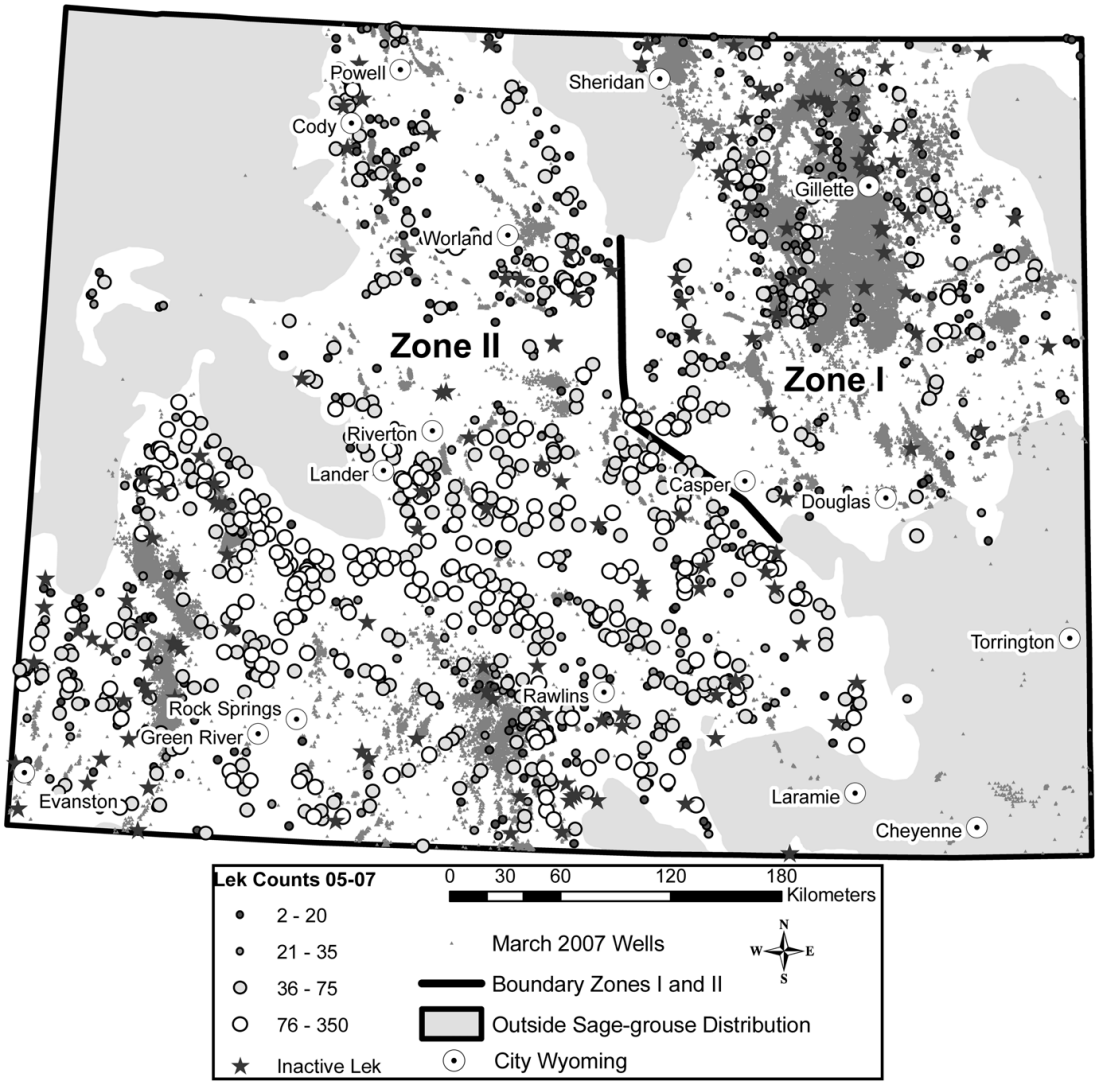
Location of sage-grouse leks and oil and gas fields in Wyoming, USA. This map displays maximum male sage-grouse counts on active leks during 2005–2007 and the location of leks that became inactive during 1999–2007. We stratified analyses by sage-grouse management zones I and II which are delineated by floristic provinces and used to group sage-grouse populations for management actions.

We adopted as a spatial framework for analyses the Western Association of Fish and Wildlife Agencies' Sage-Grouse Management Zones [31]. We stratified analyses by Management Zones I and II that divided Wyoming (Figure 1) because average lek size was larger in Zone II than I [26] and level of development was greater in Zone I than II [21]. We also incorporated a temporal component into analyses because research has shown that it takes time for cumulative impacts from development to manifest into population declines. We hypothesized that observed impacts would become more severe in time than immediately following development. High site fidelity but low survival of adult sage-grouse combined with lek avoidance by yearlings [11] resulted in a time-lag of 3–4 years between the onset of energy development and lek loss [30]. The time-lag observed by Holloran [30] in conventional gas fields in southwest Wyoming matched that for leks that became inactive 3–4 years following coal-bed natural gas development in northeast Wyoming [19]. We simulated a 4-year lag by reclassifying leks into 1 of 5 categories of development based on number of wells within 32.2 km^2^ in 2003. We also controlled for time by analyzing a subset of leks whose category of development remained the same between 2003 and 2007.

### Statistical analyses

**Figure 2:**
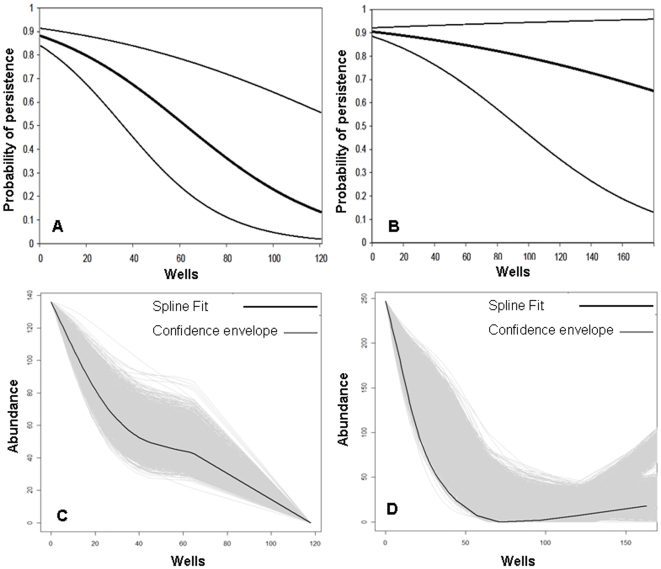
Oil and gas well density in relation to sage-grouse leks in Wyoming, USA. We used logistic regression to calculate the probability of lek persistence in relation to oil and gas well density in sage-grouse management zone I (a) and II (b) during 1999–2007. We used spline regression to compare of counts of males at leks in relation to oil and gas density in sage-grouse management zone I (c) and II (d) during 2007. Estimates incorporate a 4 year time lag since initial development.

We compared continuous well densities within 32.2 km^2^ (3.2-km radius) of a lek to rates of active/inactive leks and counts of males at leks using logistic and spline regressions. We used logistic regression [32] to analyze the relationship between well densities and whether a lek was active or inactive independently for each sage-grouse management zone. We used the mean and 95% C.I. beta coefficients to predict the probability of a lek persisting based upon observed range of well densities present in 2003 to simulates a time-lag in oil and gas development [30], [33] and we plotted rates of inactivity (Figure 2 a–b). We used a bicubic spline regression [34] to analyze abundance at leks in relation to well density independently for each sage-grouse management zone. A cross validation was run to select the optimal smoothing parameter and number of knots [35]. We implemented a Bootstrap (n = 9,999) approach to generate a confidence envelope. The axis of each graph represents the observed range and not an extrapolation to the range of the full data, since each zone was an independent model. We screened the data for non-linear relationships to rule out threshold effects before proceeding with categorical analyses. In either logistic or spline regression we did not detect relationships that would otherwise invalidate categorical analyses (Figure 2 a–d). Rather, continuous analyses warranted creation of a mitigation tool for managers that divided well densities into categories that reflect the way in which development is permitted. Linking categorical estimates with lek data to forecast anticipated impacts will provide policy makers with the information they need to weigh the biological trade-offs in permitting newly proposed or ongoing developments.

We used chi-square [36] to test for differences in rates of lek loss between category 1 (control leks with no development) and the other 4 categories of development. We first used the rate of lek loss in category 1 to determine the expected proportion of inactive leks that was not attributable to development. We calculated expected numbers of inactive leks within each category by multiplying the expected proportion of inactive leks by the total numbers of active and inactive leks within categories 2–5. We calculated chi-square statistics using expected and observed counts of inactive leks for each of 4 categories of development. We calculated the proportional change in rates of lek inactivity in relation to 4 levels of development by dividing the proportion of inactive leks in each category by the proportion of inactive leks in the control population. We put proportional increases in lek loss into context by also calculating the actual change in rate of lek loss by subtracting the observed rate within category 1 from rates within categories 2–5.

We used a 2-sample t-test [36] to test for differences in bird abundance between category 1 (control leks with no development) and 4 categories of development. We used separate variances to account for unequal variation between categories of development [37]. We calculated the ratio of standard deviations within category 1 to that within other categories. Ratios were approximately equal between categories 1 and 2 and were <2 between all other categories except category 5. We present estimates without *p*-values for category 5 because ratios were >2 in Sage-Grouse Management Zone I (ratio = 2.1) and in Zone II (5.3). Tests involving categories 1–4 conservatively ran the risk of committing a Type II error (i.e., claiming no effect of development when one really exists) because treatment categories had smaller sample sizes and variances. We did not adjust *p*-values for multiple comparisons because each individual t-test was considered a replicate test of our primary predictions. Tables of sample sizes for each test are available in supporting information files (File S1).

### Generating new hypotheses

We conducted a post-hoc analysis after findings indicated that rates of lek loss increased and bird abundance decreased with increasing levels of energy development. We mapped and inspected visually the spatial arrangement of wells for the 17 leks counted in 2007 that remained active despite having ≥40 wells within 32.2 km^2^ for ≥4 years. We did so in hope of finding a pattern that might explain a way in which sage-grouse and energy development may co-exist.

## Results

In all analyses the probability of lek persistence and abundance of males on leks declined with an increase in well density (Table 1, Figure 2 a–d). Logistic and Spline regression demonstrate that decreases to lek persistence were more severe in sage-grouse management zone I (Figure 2a versus 2b) and decreases to abundance were more severe in management zone II (Figure 2c versus 2d). Categorical analyses also document that rate of lek loss was greater in Management Zone I than Zone II and declines in males at affected leks were greatest in Zone II (Table 1, Figure 1). Rate of lek loss increased from 2 to 5 times that outside of development in Zone I when densities exceeded 40 wells per 32.2 km^2^ (Table 1), a level of development that is commonly permitted on public lands (e.g., 32 ha or 80 ac well spacing). At this level of development in Zone II, the increased rate of lek loss was 3 times that outside of development and bird abundance at remaining leks inside development declined by −59% (Table 1). Background rates of lek inactivity outside development were 12% and 9% in Management Zones I and II respectively. Category 2 at 1–12 wells per 32.2 km^2^ (∼1 well per cadastral section of land [259 ha or 640 ac]) represented a level of development within which impacts to leks were indiscernible (Table 1). Beyond this threshold of development lek loss and declines in birds at remaining leks increased regardless of management zone (Table 1). Continuous analyses using logistic and spline regression support results of categorical analyses used to create a mitigation tool.

**Table 1:** Increased risk of lek loss, resulting decline in active leks (%), decline in males on remaining active leks (%) and resulting chi-square tests between control leks with no development and those inside of 4 categories of increasing oil and gas development, by Sage-Grouse Management Zones I and II 1997–2007, Wyoming, USA (31; Figure 1).

Number of Wells per 32.2 km^2^ (Well Spacing)^a^	Increased Risk of Lek Loss^b^	Resulting Decline in Active Leks (%)^b^^,^ c	Decline in Males (%) on Remaining Active Leks^b^^,^ c ^,^ d
Management Zone I			
1–12 (259 ha; 640 ac)	1.1 (*p*>0.25)	−0.7 (*p*>0.25)	−2.1% (*p* = 0.43)
13–39 (65 ha; 160 ac)	2.0 (*p*<0.02)	−11.5 (*p*<0.02)	−31.4% (*p*<0.01)
40–100 (32 ha; 80 ac)	5.1 (*p*<0.01)	−47.2 (*p*<0.01)	−32.6% (*p* = 0.13)
101–199 (16 ha; 40 ac)	5.7 (NA)^f^	−55.1 (NA)	−77.3% (NA)
Management Zone II			
1–12 (259 ha; 640 ac)	1.1 (*p*<0.05)	−1.0 (*p*>0.25)	0.1% (*p* = 0.50)
13–39 (65 ha; 160 ac)	2.4 (*p*<0.01)	−12.1 (*p*<0.01)	−55.5% (*p*<0.01)
40–100 (32 ha; 80 ac)	2.8 (*p*<0.10)	−16.1 (*p*<0.05)	−59.0% (*p*<0.01)
101–199 (16 ha; 40 ac)			−69.5% (NA)

a Number of producing oil and gas wells within a 32.2 km^2^ (3.2-km radius) of a lek and average spacing between adjacent wells (ha and ac).

b Increased risk of lek loss associated with increasing levels of development. For example, risk of lek loss was 5.1 times greater inside than outside of development in Zone I when densities were 40–100 wells per 32.2 km^2^.

c Estimates include a time-lag affect because it takes 4 years for impacts to manifest into population declines (File S1). Estimates also are adjusted for background losses not attributable energy development.

d Increasing proportion of leks that go inactive with increasing levels of development.

e Declines in active leks (%) and in males on remaining active leks (%) can be applied to assess trade-offs of newly proposed or ongoing development. Resulting declines in bird numbers form the biological basis for negotiating offsets.

f Chi-square test not performed if sample size <5.

A time-lagged response showed higher rates of lek loss and steeper declines in bird abundance 4 years after than immediately following development. Time lag effects in bird abundance were most apparent at lower levels of development (13–39 wells) whereas rate of lek loss was most affected at higher levels of development (40–100 wells) (File S1). The largest time-lag effect on lek persistence was in Zone I where rate of lek loss initially doubled and after 4 years was 5 times that outside of development (Table 1). This rate corresponded to a 47–55% increase in lek loss when development was ≥40 wells within 32.2 km^2^ (Table 1). The greatest time-lag effect on bird abundance was in Zone II where male counts on affected leks declined by 55.5% (Table 1). Impacts remained constant after leks that switched disturbance categories between 2003 and 2007 were removed from analyses (File S1).

**Figure 3:**
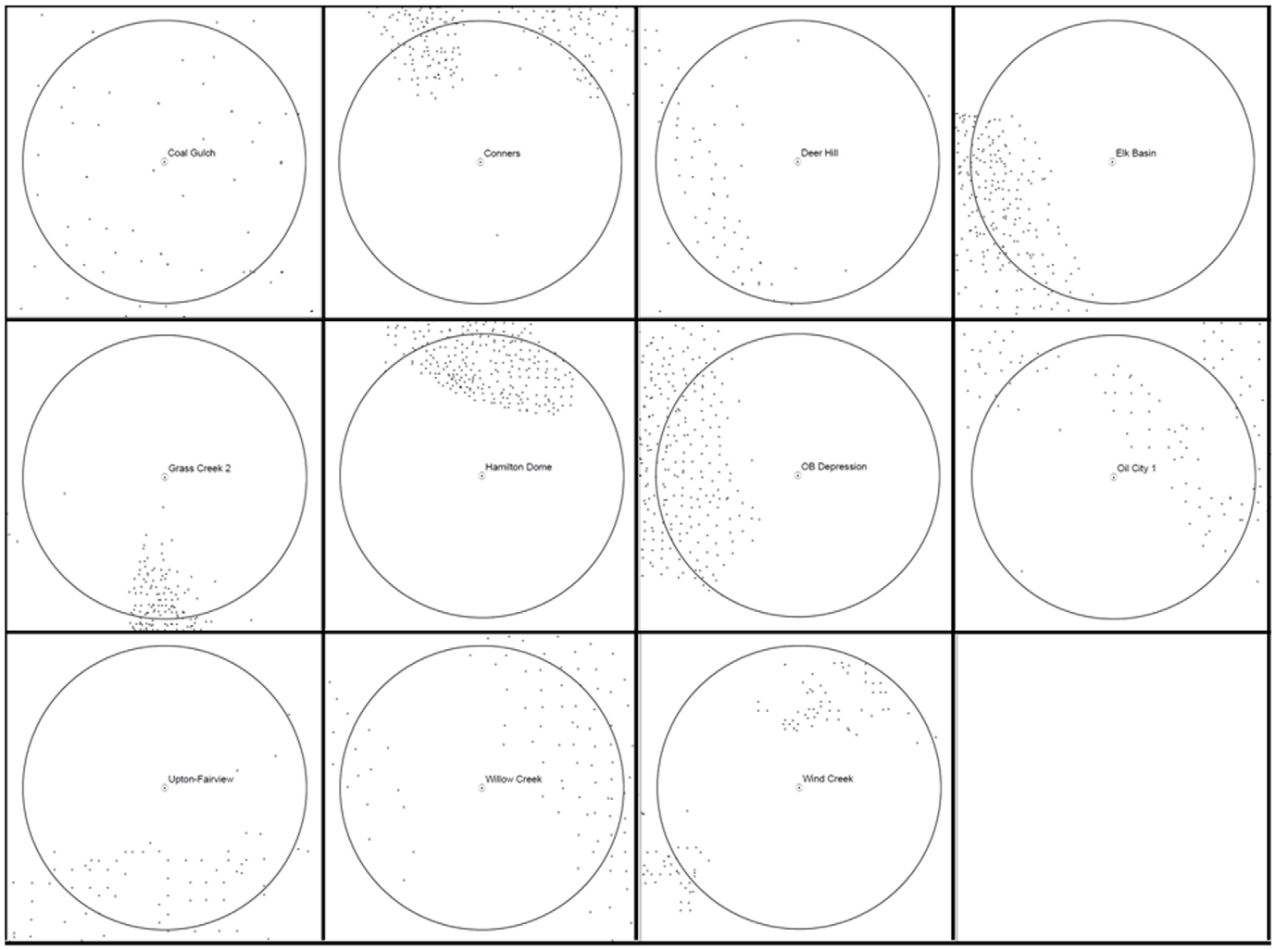
Spatial arrangement of oil and gas wells around active leks in Wyoming, USA. Clumping of oil and gas wells which maintained open areas around the lek was evident at 11 of 17 leks that remained active with ≥40 wells within 32.2 km^2^. Circles represent a 3.2 km buffer around a lek (white dot) and small black dots are locations of oil and gas wells.

In Wyoming 15.1% of active leks (*n* = 156 of 1,035) had >12 wells within 32.2 km^2^ in 2007, of which 17 (10.9%) remained active with ≥40 wells within 32.2 km^2^ for ≥4 years. Bird abundance was 55% lower than the state-wide average at these 17 leks that remained active despite high development. A post-hoc visual inspection showed that wells were clustered in a high density pattern that maintained open areas within 32.2 km^2^ for 64.7% (11 of 17) of these leks (Figure 3). Further evaluation of Oil City 1 lek showed that it was 1 of 4 leks that remained active within Management Zone I despite high development (40–100 wells for ≥4 years). A maximum count of 40 males at Oil City 1 lek in 2007 was 1.47 times higher compared to leks outside of development in Management Zone I. If Oil City 1 was removed from analyses declines in abundance for leks with 40–100 wells for ≥4 years doubled (from −18.2 to −32.6% [*p* = 0.125; Table 1] and from −23.2 to −46.5% [*p* = 0.030; File S1]).

## Discussion

The ability to forecast impacts is critical to making informed decisions, especially when land use plans call for adaptive management of multiple resource values including sage-grouse [38]. The value of these findings is in weighing the biological consequences of ongoing and future anticipated developments in different landscapes. Impacts were evident at large spatial scales (Figure 1) and across a wide range of development intensities (Table 1) such that they can be used as the best estimate of the residual impacts of energy development on sage-grouse populations. Assessments should be conducted using estimates of impacts from the appropriate management zone (Table 1). Continuous analyses highlight impacts between management zones show similar patterns, but vary in actual probability of persistence and impacts to abundance in relation to well density (Fig 2 a–d). For example, managers contemplating lease sales that authorize full-field development (80 ac spacing) in Management Zone I can anticipate resulting impacts by cutting in half (−47.2%) the number of remaining leks and reducing by a third (−32.6%) the number of birds on remaining active leks (Table 1). Likewise, managers judging whether to permit additional wells in existing fields can forecast further impacts by updating maps to identify leks that move into higher disturbance categories. This information can also be linked to future development scenarios to anticipate consequences of different land-use planning options at regional scales.

Statewide analyses in Wyoming showed that impacts to sage-grouse populations from energy development were severe at oil and gas well densities commonly permitted on public lands (Table 1). Impacts reported here incorporated known time-lags, stratified findings by management zone, made complete use of the best available data and compared affected and unaffected (i.e., control) leks. Control leks were integral to findings because without them we would have missed impacts at a level of development below that which is commonly permitted on public lands. Impacts were evident inside of energy developments despite a 15% increase in the overall number of displaying males at active leks in Wyoming [39]. Findings reiterated the importance of time-lags [30], [19] as evidenced by greater impacts 4 years after rather than immediately following development. The time-lag effect reported here is similar to that in a recent meta-analysis of impacts of wind energy on bird abundance [40]. Declines in Zone II where drilling is underway in earnest are especially disconcerting because affected leks are some of the largest in the remaining range of the species [26], [22]. Differences in estimates between zones could be related to initial lek size (size of control leks in Zones I [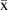 = 27.2, SE = 2.6] and II [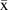 = 47.8, SE = 1.9]), initial habitat quality prior to development, and overall extent of development within zones (Figure 1). Research of radio-marked sage-grouse has shown lower survival of adult female sage-grouse resulting in population level declines [30], but has also shown increased mortality of yearling sage-grouse and yearling avoidance of leks inside development [11]. Until future research demonstrates that avoidance of energy development which reduces the distribution of sage-grouse does not result in population declines from density dependence, competition, or displacement into poor-quality habitats which lowers survival or reproduction among displaced birds [17], [41], [42] avoidance is not proven mitigation.

Energy independence is an issue of national security in the U.S. As this nation increases domestic production to reduce its dependency on foreign sources one thing is clear—increased wildlife impacts are inevitable. Policies to reduce impacts should include all aspects of the mitigation hierarchy (avoid, minimize, restore and offset). Biodiversity offsets are a necessary part of conservation after preceding steps in the mitigation hierarchy have been exhausted. Offsets represent a partnership between industry and conservation and provide a proactive solution for accommodating development of domestic energy resources. The concept of offsets is a part of the culture of mining and electrical transmission industries but is still a relatively new and largely unexplored topic in other energy development sectors. The National Environmental Policy Act or ‘NEPA process’ readily provides for off-site mitigation despite the current novelty of biodiversity offsets in the energy arena. Most importantly, offsets provide a partial solution for funding large-scale conservation while the option to do so is still available.

Our findings show how birds rather than habitat should be used as a common and biologically-based currency for estimating sufficiency of offsets. Offsetting other forms of risk using birds as the currency can be implemented immediately by affording industry the opportunity to add biodiversity costs to their balance sheets through off-site mitigation. Offsets may permanently protect a population of equal or greater number of birds from future population losses that would have occurred if offsets did not mitigate other forms of risk. For example, conservation easements could be purchased to protect sagebrush dominated ranch lands having a high risk of agriculture conversion from tillage agriculture. Other forms of risks to sage-grouse across the range that could be mitigated vary and include buying back oil and gas development rights and juniper encroachment treatments. Monetary costs of protective conservation easements can be high. Cumulative easement costs were estimated at $47–90 million, with average costs ranging from $600–$1,000/acre, to mitigate multi-state transmission corridor in Idaho and Montana for sage-grouse [43]. Using a biological currency in mitigation provides a monetary incentive for industry to proactively implement conservation by protecting an equal or greater number of birds in priority landscapes [22]. Focusing offsets in areas of high biological value also lowers offset costs because mitigation will benefit more birds per unit area. Incorporating incentive structures into conservation strategies helps align interests of industry, landowners, and conservationists [44].

Enhancing habitats to increase sage-grouse populations within priority landscapes is a more complex but equally important step in offsetting impacts. This recommendation may be the most difficult to implement because few long-term and replicated experiments showing a positive population response to management have been conducted. We recommend additional field-based experimental research to identify the most effective and least expensive ways to increase populations. The high cost of protective off-site mitigation measures may serve as a catalyst to fund research on how to enhance habitats to increase sage-grouse populations. Until then we recommend habitat manipulations focus on restoring sagebrush and fostering strategies that enhance grass height and forbs to align with food and cover requirements outlined in the sage-grouse management guidelines [45]. Research on grazing and invasive species management are high priorities for sage-grouse conservation [46].

The simplest and most cost effective first step in conservation is to halt the large-scale actions that further reduce or eliminate the largest populations in the best remaining landscapes [16], [22]. Most states and federal agencies responsible for managing sage-grouse populations and their habitats have mapped the locations of their large and intact priority landscapes that support core populations. Using documented sage-grouse impacts as a mitigation currency provides the science necessary to backup mitigation decisions when avoidance is not possible. Our post-hoc investigation of well clustering shows that maintaining some open areas for nesting may help keep a few small leks active inside of developments (Figure 3). Having small, but active leks may increase our ability to restore populations following development because strong site fidelity in sage-grouse [47], [48] makes natural re-colonization slow and past precedence has documented that translocations into areas with no resident populations are unlikely to succeed [49], [50]. We recommend updating estimates provided in this mitigation framework as new information on mechanisms of population declines are discovered, as affects of other covariates such initial habitat quality prior to development become available on a state-wide scale, and as hypotheses such as well clustering are evaluated. Until then, our analyses makes complete use of the best available data by comparing affected and unaffected (i.e., control) leks to provide a biologically-based and common currency for estimating sufficiency of offsets.

## Supporting Information

File S1 (0.13 MB DOC)
