# Supplementary material for: A Currency for Offsetting Energy Development Impacts: Horse-Trading Sage-Grouse on the Open Market
Source: PLoS One. 2010 Apr 28;5(4):e10339. doi: 10.1371/journal.pone.0010339 (PMC2860982; doi:10.1371/journal.pone.0010339)
Supplement: File S1 — (0.13 MB DOC) [file pone.0010339.s001.doc]

Doherty et al. Supporting Information File S1

Appendix S1. Sample sizes of leks within each treatment categories for lek inactivity in Wyoming 1997-2007^a^

| Number of wells ^b^ | Active  2007 | Inactive 2007 | Active 2007 4-yr time-lag ^c^ | Inactive 2007 4-yr time-lag ^c^ | Active 2007 4-yr time-lag ^d^ | Inactive 2007 4-yr time-lag ^d^ |
| --- | --- | --- | --- | --- | --- | --- |
| 0 = Control | 766 | 80 | 835 | 86 | 744 | 79 |
| 1-12 | 257 | 24 | 260 | 32 | 181 | 18 |
| 13-39 | 108 | 22 | 74 | 21 | 52 | 15 |
| 40-100 | 48 | 19 | 16 | 13 | 12 | 10 |
| 101-199 | 10 | 9 | 5 | 2 | 4 | 2 |
| Total | 1189^e^ | 154 | 1190 | 154 | 1002 | 125 |

^a^ We stratified analyses by Sage-grouse Management Zones I and II to reflect differences in average lek size and intensity of development [26].

^b^ We quantified intensity of development as number of energy wells within 32.2 km^2^ of a lek (19).

^c^ We incorporated a time-lag into analyses because it takes 4-yrs for cumulative impacts from development to manifest into population declines [19, 30].

^d^ We removed leks that switched categories between 2003 and 2007 to control for confounding effect of increasing intensity of development.

^e^ We lek with greater than 199 wells was removed from the analyses.

Appendix S2. Sample sizes of leks within each treatment categories for lek inactivity in sage-grouse management zone I, Wyoming 1997-2007^a^.

| Number of wells ^b^ | Active  2007 | Inactive 2007 | Active 2007 4-yr time-lag ^c^ | Inactive 2007 4-yr time-lag ^c^ | Active 2007 4-yr time-lag ^d^ | Inactive 2007 4-yr time-lag ^d^ |
| --- | --- | --- | --- | --- | --- | --- |
| 0 = Control | 104 | 15 | 137 | 18 | 103 | 15 |
| 1-12 | 81 | 8 | 100 | 14 | 61 | 6 |
| 13-39 | 64 | 10 | 40 | 12 | 27 | 7 |
| 40-100 | 30 | 15 | 7 | 10 | 4 | 7 |
| 101-199 | 8 | 8 | 1 | 2 | na | 2 |
| Total | 284^e^ | 56 | 285 | 56 | 1002 | 125 |

^a^ We stratified analyses by Sage-grouse Management Zones I and II to reflect differences in average lek size and intensity of development [26].

^b^ We quantified intensity of development as number of energy wells within 32.2 km^2^ of a lek (19).

^c^ We incorporated a time-lag into analyses because it takes 4-yrs for cumulative impacts from development to manifest into population declines [19, 30].

^d^ We removed leks that switched categories between 2003 and 2007 to control for confounding effect of increasing intensity of development.

^e^ We lek with greater than 199 wells was removed from the analyses.

Appendix S3. Sample sizes of leks within each treatment categories for lek inactivity in sage-grouse management zone II, Wyoming 1997-2007^a^.

| Number of wells ^b^ | Active  2007 | Inactive 2007 | Active 2007 4-yr time-lag ^c^ | Inactive 2007 4-yr time-lag ^c^ | Active 2007 4-yr time-lag ^d^ | Inactive 2007 4-yr time-lag ^d^ |
| --- | --- | --- | --- | --- | --- | --- |
| 0 = Control | 662 | 65 | 698 | 68 | 641 | 64 |
| 1-12 | 176 | 16 | 160 | 18 | 120 | 12 |
| 13-39 | 44 | 12 | 34 | 9 | 25 | 8 |
| 40-100 | 18 | 4 | 9 | 3 | 8 | 3 |
| 101-199 | 5 | 1 | 4 | na | 4 | na |
| Total | 905 | 98 | 905 | 98 | 1002 | 125 |

^a^ We stratified analyses by Sage-grouse Management Zones I and II to reflect differences in average lek size and intensity of development [26].

^b^ We quantified intensity of development as number of energy wells within 32.2 km^2^ of a lek (19).

^c^ We incorporated a time-lag into analyses because it takes 4-yrs for cumulative impacts from development to manifest into population declines [19, 30].

^d^ We removed leks that switched categories between 2003 and 2007 to control for confounding effect of increasing intensity of development.

^e^ We lek with greater than 199 wells was removed from the analyses.

Appendix S4. Sample Sizes of leks surveyed in Wyoming during 2007 with ≥ 2 male sage-grouse (*n* = 1035) used for abundance tests blocked by sage-grouse management zones I and II.

| Sage-grouse Management Zone I ^a^ | | | |
| --- | --- | --- | --- |
| Number of wells ^b^ | Active 2007 | 4-yr time-lag ^c^ | 4-yr time-lag ^d^ |
| 0 = Control | 94 | 129 | 93 |
| 1-12 | 74 | 91 | 55 |
| 13-39 | 63 | 39 | 27 |
| 40-100 | 29 | 6 | 4 |
| 101-199 | 4 | 1 | 1 |
| Total | 264^e^ | 265 | 180 |
| Sage-grouse Management Zone II ^a^ | | | |
| Number of wells ^b^ | Active 2007 | 4-yr time-lag ^c^ | 4-yr time-lag ^d^ |
| 0 = Control | 556 | 618 | 538 |
| 1-12 | 155 | 112 | 102 |
| 13-39 | 39 | 31 | 22 |
| 40-100 | 16 | 7 | 6 |
| 101-199 | 4 | 3 | 3 |
| Total | 770 | 770 | 671 |

^a^ We stratified analyses by Sage-grouse Management Zones I and II to reflect differences in average lek size and intensity of development [26].

^b^ We quantified intensity of development as number of energy wells within 32.2 km^2^ of a lek (19).

^c^ We incorporated a time-lag into analyses because it takes 4-yrs for cumulative impacts from development to manifest into population declines [19, 30].

^d^ We removed leks that switched categories between 2003 and 2007 to control for confounding effect of increasing intensity of development.

^e^ We lek with greater than 199 wells was removed from the analyses.

Appendix S5. Effect of a 4-year time-lag on the risk of lek loss and resulting chi-square tests between control leks with no development and those inside of 4 categories of increasing oil and gas development, stratified by Sage-Grouse Management Zones I and II 1997-2007, Wyoming, USA (31; Figure 1).

| Number of Wells per 32.2 km^2^  (Well Spacing)^a^ | No time-lag | | 4-yr Time-lag  Applied to All Leks | Lag Applied to Leks that Did Not Change Disturbance Categories During Time-lag Period^b^ |
| --- | --- | --- | --- | --- |
| Management Zone I | | | | |
| 1-12 (259 ha; 640 ac) | | 0.71 (*p* > 0.25) | 1.06 (*p* > 0.25) | 0.70 (*p* > 0.25) |
| 13-39 (65 ha; 160 ac) | | 1.07 (*p* > 0.25) | 2.00 (*p* < 0.02) | 1.61 (*p* < 0.20) |
| 40-100 (32 ha; 80 ac) | | 2.64 (*p* < 0.01) | 5.07 (*p* < 0.01) | 5.00 (*p* < 0.01) |
| 101-199 (16 ha; 40 ac) | | 4.88 (*p* < 0.01) | 5.74 (NA)^c^ | 7.87 (NA) |
| Management Zone II | | | | |
| 1-12 (259 ha; 640 ac) | | 0.93 (*p* > 0.25) | 1.14 (*p* < 0.05) | 1.00 (*p* > 0.25) |
| 13-39 (65 ha; 160 ac) | | 2.40 (*p* < 0.01) | 2.36 (*p* < 0.01) | 2.67 (*p* < 0.01) |
| 40-100 (32 ha; 80 ac) | | 2.03 (*p* < 0.15) | 2.82 (*p* < 0.10) | 3.00 (*p* < 0.05) |
| 101-199 (16 ha; 40 ac) | | 1.86 (NA) |  |  |

^a^ Number of producing oil and gas wells within a 32.2 km^2^ (3.2-km radius) of a lek and average spacing between adjacent wells (ha and ac).

^b^ We removed leks that switched categories between 2003 and 2007 to test for possible confounding effects of increasing levels of development through time.

^c^ Chi-square tests were not performed if sample sizes were < 5.

Appendix S6. Effect of a 4-year time-lag on declines in males at remaining active leks and resulting t-tests between control leks with no development and those inside of 4 categories of increasing oil and gas development, stratified by Sage-Grouse Management Zones I and II 1997-2007, Wyoming, USA (31; Figure 1).

| Number of Wells per 32.2 km^2^  (Well Spacing)^a^ | No time-lag | | 4-yr Time-lag  Applied to All Leks | | Lag Applied to Leks that Did Not Change Disturbance Categories During Time-lag Period^b^ |
| --- | --- | --- | --- | --- | --- |
| Management Zone I | | | | | |
| 1-12 (259 ha; 640 ac) | | -2.5% (*p* = 0.43) | | -2.1% (*p* = 0.43) | 6.9% (*p* = 0.71) |
| 13-39 (65 ha; 160 ac) | | -17.0% (*p* = 0.09) | | -31.4% (*p* < 0.01) | -29.2% (*p* = 0.02) |
| 40-100 (32 ha; 80 ac) | | -41.4% (*p* < 0.01) | | -32.6% (*p* = 0.13) | -46.5% (*p* = 0.02) |
| 101-199 (16 ha; 40 ac) | | -34.4% (NA)^c^ | | -77.3% (NA) | -78.3% (NA) |
| Management Zone II | | | | | |
| 1-12 (259 ha; 640 ac) | | -8.0% (*p* = 0.11) | | 0.1% (*p* = 0.50) | -2.9% (*p* = 0.64) |
| 13-39 (65 ha; 160 ac) | | -35.9% (*p* < 0.01) | | -55.5% (*p* < 0.01) | -58.8% (*p* < 0.01) |
| 40-100 (32 ha; 80 ac) | | -55.1% (*p* < 0.01) | | -59.0% (*p* < 0.01) | -58.6% (*p* < 0.01) |
| 101-199 (16 ha; 40 ac) | | -68.5% (NA) | | -69.5% (NA) | -69.4% (NA) |

^a^ Number of producing oil and gas wells within a 32.2 km^2^ (3.2-km radius) of a lek and average spacing between adjacent wells (ha and ac).

^b^ We removed leks that switched categories between 2003 and 2007 to test for possible confounding effects of increasing levels of development.

^c^ T-test not performed if sample size in treatment and control was < 5 or if ratio of standard deviation between groups was > 2.
